# Targeted Delivery for Cardiac Regeneration: Comparison of Intra-coronary Infusion and Intra-myocardial Injection in Porcine Hearts

**DOI:** 10.3389/fcvm.2022.833335

**Published:** 2022-02-10

**Authors:** Andrew M. Vekstein, David C. Wendell, Sophia DeLuca, Ruorong Yan, Yifan Chen, Muath Bishawi, Garth W. Devlin, Aravind Asokan, Kenneth D. Poss, Dawn E. Bowles, Adam R. Williams, Nenad Bursac

**Affiliations:** ^1^Division of Cardiovascular and Thoracic Surgery, Department of Surgery, Duke University Medical Center, Durham, NC, United States; ^2^Duke Cardiovascular Magnetic Resonance Center, Duke University Medical Center, Durham, NC, United States; ^3^Department of Biomedical Engineering, Duke University, Durham, NC, United States; ^4^Department of Cell Biology, Duke Regeneration Center, Duke University, Durham, NC, United States; ^5^Department of Surgery, Duke University Medical Center, Durham, NC, United States; ^6^Department of Molecular Genetics and Microbiology, Duke University Medical Center, Durham, NC, United States; ^7^Department of Surgery, Surgical Sciences, Duke University Medical Center, Durham, NC, United States

**Keywords:** heart, gene therapy, iron oxide, regeneration, magnetic resonance imaging

## Abstract

**Background:**

The optimal delivery route to enhance effectiveness of regenerative therapeutics to the human heart is poorly understood. Direct intra-myocardial (IM) injection is the gold standard, however, it is relatively invasive. We thus compared targeted IM against less invasive, catheter-based intra-coronary (IC) delivery to porcine myocardium for the acute retention of nanoparticles using cardiac magnetic resonance (CMR) imaging and viral vector transduction using qPCR.

**Methods:**

Ferumoxytol iron oxide (IO) nanoparticles (5 ml) were administered to Yorkshire swine (*n* = 13) by: (1) IM via thoracotomy, (2) catheter-based IC balloon-occlusion (BO) with infusion into the distal left anterior descending (LAD) coronary artery, (3) IC perforated side-wall (SW) infusion into the LAD, or (4) non-selective IC via left main (LM) coronary artery infusion. Hearts were harvested and imaged using at 3T whole-body MRI scanner. In separate Yorkshire swine (*n* = 13), an adeno-associated virus (AAV) vector was similarly delivered, tissue harvested 4–6 weeks later, and viral DNA quantified from predefined areas at risk (apical LV/RV) vs. not at risk in a potential mid-LAD infarct model. Results were analyzed using pairwise Student's *t*-test.

**Results:**

IM delivery yielded the highest IO retention (16.0 ± 4.6% of left ventricular volume). Of the IC approaches, BO showed the highest IO retention (8.7 ± 2.2% vs. SW = 5.5 ± 4.9% and LM = 0%) and yielded consistent uptake in the porcine distal LAD territory, including the apical septum, LV, and RV. IM delivery was limited to the apex and anterior wall, without septal retention. For the AAV delivery, the BO was most efficient in the at risk territory (Risk: BO = 6.0 × 10^−9^, IM = 1.4 × 10^−9^, LM = 3.2 × 10^−10^ viral copies per μg genomic DNA) while all delivery routes were comparable in the non-risk territory (BO = 1.7 × 10^−9^, IM = 8.9 × 10^−10^, LM = 1.2 × 10^−9^).

**Conclusions:**

Direct IM injection has the highest local retention, while IC delivery with balloon occlusion and distal infusion is the most effective IC delivery technique to target therapeutics to a heart territory most in risk from an infarct.

## Introduction

Cardiac gene and stem cell therapies present promising avenues to stimulate myocardial recovery after an infarct or from non-ischemic cardiomyopathies (1–3). Various methods to deliver cells and genes into the myocardium have been developed (4–6). Specifically, intra-myocardial (IM) injection involves direct epicardial or catheter-based trans-endocardial delivery of the therapeutic into the interstitial space of the heart wall (7). Intra-coronary (IC) catheter-based delivery has been performed with either non-selective infusion into the right or left coronary artery or selective infusion into a single branch vessel with proximal balloon occlusion to slow flow that may wash out the delivered therapeutic (8, 9). Retrograde delivery of the therapeutic with occlusion of the coronary sinus or coronary venous branch has also been utilized in several studies (10–12). Finally, pericardial dwelling has been used to facilitate slow uptake of the therapeutic to the epicardium (13). Among these delivery strategies, trans-epicardial IM techniques are limited by the invasiveness of the approach to reach the heart (most commonly thoracotomy), while the IC options may be limited by impermeability of the vascular endothelium and rapid washout of the therapeutic (6). Thus, the optimal delivery technique to enhance retention of the therapeutic in the heart, and ultimately the effectiveness of therapy, while minimizing risk to the patient, has not been yet established and will likely vary among different applications (4–6).

In the context of gene therapy for heart failure or myocardial infarction (MI), adeno-associated virus (AAV) has been a vector of choice for stable gene delivery to cardiomyocytes (6). AAV vectors are relatively small in size (25 nm), generally considered non-pathogenic, and depending on a serotype used, they can be effectively delivered to cardiomyocytes either by IM or IC delivery. In particular, AAV serotype 9 (AAV9) has been gaining popularity for cardiac gene therapy due to its natural tropism for cardiomyocytes and the ability to effectively penetrate the endothelial barrier. However, the optimal delivery route for AAV-based therapies in the human heart is still a matter of debate (14). As such, the objective of this study was to compare IM, non-selective IC, and targeted balloon occlusion IC delivery techniques in porcine myocardium as a physiologically-relevant model of human heart. Specifically, for the studied delivery techniques, we assessed: (1) the retention of hyperparamagnetic nanoparticles with similar size to AAV vectors using cardiac magnetic resonance imaging (CMR) in an acute delivery model and (2) AAV9 transduction efficacy using qPCR in separate animals undergoing longer-term survival.

## Methods

### Porcine Model

**Figure 1:**
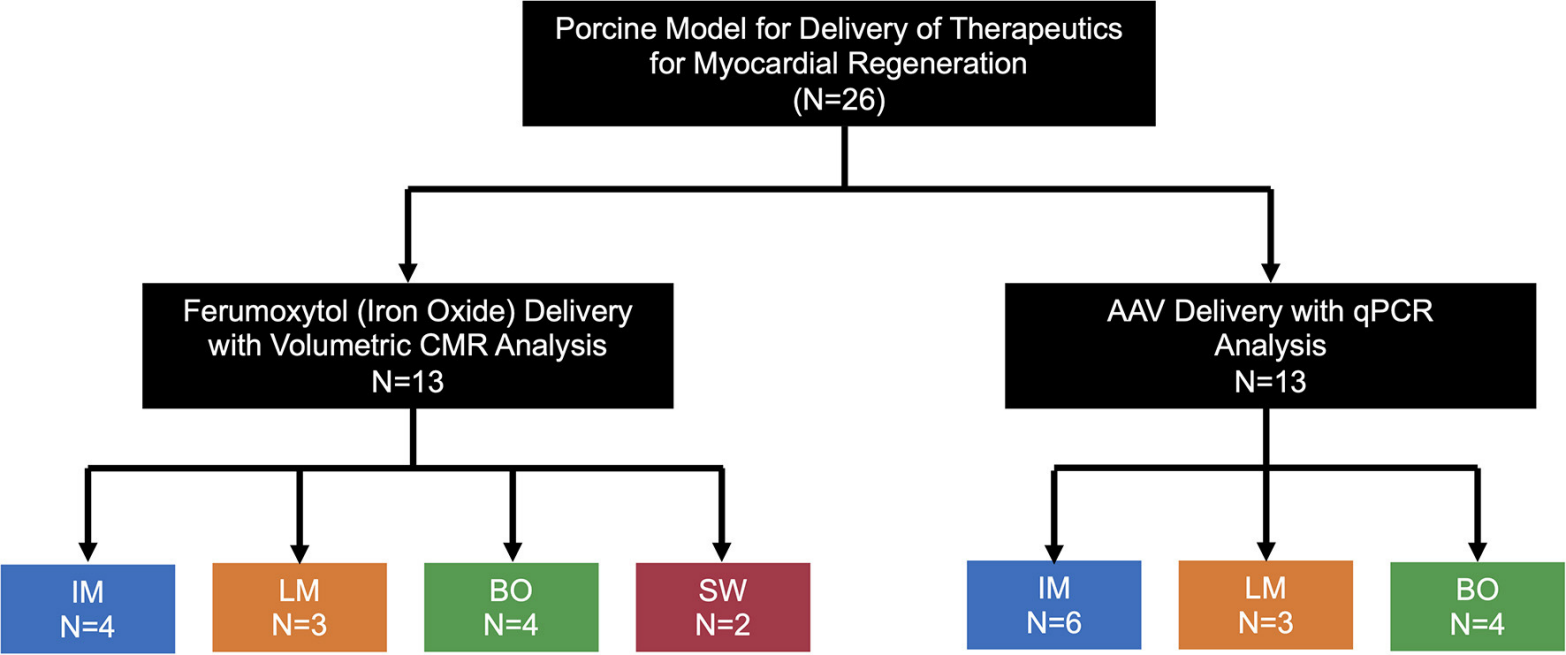
Schematics of the experimental design. Pigs were allocated to the IO or AAV substudy (*n* = 13 each) with various delivery techniques tested. IM, Intramyocardial; LM, Left main coronary artery injection; BO, Balloon Occlusion; SW, Sidewall.

All animal experiments have been approved by the Duke University Institutional Animal Care and Use Committee (IACUC). Myocardial therapeutic delivery techniques were compared in Yorkshire swine (30–40 kg, Wesley Looper Pig Farm, Granite Falls, NC) in two study arms, by tracking *in-vivo* delivery of: (1) iron oxide nanoparticles 25 nm in size (IO, ferumoxytol) (*n* = 13) or (2) adeno-associated virus serotype 9 (AAV) (*n* = 13) (Figure 1). AAV experiments were a preceded by a myocardial infarction (MI) and involved use of multiple transgenes. All procedures in swine were performed under deep anesthesia using ketamine 4 mg/kg intramuscular, midazolam 0.5 mg/kg intramuscular, and isoflurane 0.5–3.0% inhalation via endotracheal intubation. Antiarrhythmic therapy included lidocaine 3 mg/kg bolus followed by a continuous infusion at 2 mg/min. Delivery of IO or AAV was performed via intramyocardial injection (IM), direct left-main IC infusion (LM), balloon occlusion intracoronary IC infusion in the left anterior descending (LAD) coronary artery (BO), or sidewall catheter IC infusion in the LAD (SW). Hearts were harvested for *ex-vivo* cardiac magnetic resonance imaging (CMR) tracking of IO, or for assessment of AAV vector transduction. In order to assess the degree of myocardial injury specific to a delivery technique, high sensitivity troponin I (Marshfield Labs, Marshfield, WI) levels in blood were assessed immediately after induction of anesthesia for baseline levels and 15 min after completion of IO delivery, immediately prior to the heart harvest. The relatively short 15 min post-IO delivery time point for troponin level assessment was selected to minimize IO washout from the heart. The AAV-injected animals were not included in the troponin analysis to avoid confounding results from an MI injury prior to AAV delivery. We favored troponin I based on previous studies of biomarkers of myocardial ischemia in porcine models (15) and chose not to perform creatinine kinase (CK-MB) assessment due to possible confounding results from intercostal muscle injury induced by the thoracotomy performed for IM injection.

### Delivery Technique: Intramyocardial Injection (IM)

**Figure 2:**
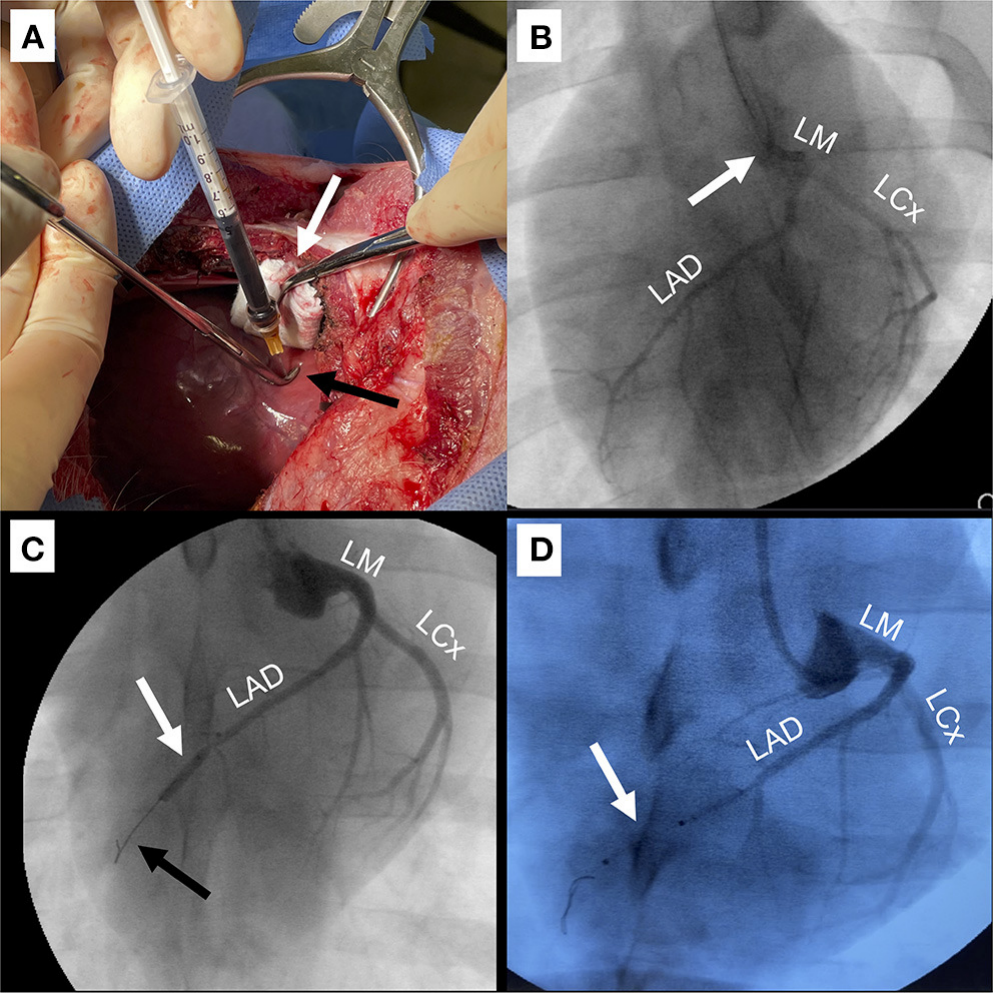
Techniques for delivery of IO/AAV. **(A)** Intramyocardial injection (IM) through anterior right thoracotomy. The apex was retracted to the right to expose the anterior and apical regions (white arrow) and a 25-gauge 5/8″ needle in a right-angled clamp (black arrow) was used to mediate depth of injection. **(B)** Direct left main coronary artery infusion (LM). Coronary angiographic catheter (white arrow) was positioned in the LM. Infused radiographic contrast marks targeted coronary arteries including the left circumflex (LCx) and left anterior descending (LAD). **(C)** Balloon occlusion with distal infusion (BO). Angioplasty balloon was positioned in the mid-LAD (white arrow) and inflated for 60–90 s to occlude distal flow (black arrow), during which IO/AAV was infused through the distal tip of the balloon catheter. **(D)** Perforated sidewall balloon infusion (SW). IO/AAV was infused through the sidewall of a non-occlusive perforated balloon (white arrow) positioned to extend from the mid- to distal-LAD.

Four swine in the IO study arm and six swine in the AAV study arm underwent IM injection (Figure 2A). For this approach, a 5–6 cm anterior right thoracotomy was performed in the fifth intercostal space. Contrary to human anatomy, the porcine heart is vertically oriented in the mediastinum due to the pig's concave sternum; thus, a right sided approach yields best access to the apex and distal LAD territory in the pig. The lung was compressed with a moist gauze to expose the pericardium, which was opened longitudinally with scissors with care to remain at least 1 cm anterior to the phrenic nerve. The anterior pericardium was tacked to the skin with 2-0 silk stitches. For optimal IM delivery of therapeutics, injection territory was exposed and stabilized as follows. A dry gauze within the tip of a clamp was used to stabilize and expose the apex of the heart. The LAD was identified coursing toward the apex and avoided in injection sites. Once the heart was stabilized, a 25 gauge 5/8″ needle was used to inject 0.5 ml aliquots of iron oxide in 8–10 separate sites in the anterolateral wall and apex. In order to mediate needle depth to 2–3 mm (within the myocardium), the needle tip was placed within a right-angle clamp (Figure 2A), which served as a bumper on the epicardial surface. Hemostasis was achieved by direct pressure of the injection site by compressing a clamp with gauze in the tip at the injection site for 10–20 s. At the completion of injections, all injection sites were inspected for potential bleeding and direct pressure applied if active bleeding was identified. A chest tube was placed through a counterincision and connected to wall suction. The incision was closed in layers with absorbable suture and skin glue was applied. Finally, the chest tube was removed prior to extubation.

### Delivery Technique: Carotid Arterial Access for Intracoronary Infusion

A surgical cutdown was performed through a vertical paratracheal incision and the common carotid artery dissected free over several centimeters and encircled with a vessel loop. Heparin 200–300 units/kg was administered intravenously. The artery was then accessed with a 21-gauge micropuncture introducer needle and 5-french micropuncture catheter was advanced over a wire, which was subsequently upsized to a 6-french vascular sheath.

### Delivery Technique: Direct Left Main Intracoronary Infusion (LM)

Three swine underwent LM infusion in both the IO and AAV study arms (Figure 2B, Supplementary Video 1). LM delivery was performed with standard coronary angiography techniques using fluoroscopic “C-arm” guidance. Specifically, a 6-french Judkins Right (JR) 4 coronary diagnostic catheter (Cordis, Santa Clara, CA) was advanced over a 0.035” guidewire from the carotid artery sheath into the aortic root. JR catheters provide the appropriate curvature for accessing the left main (LM) coronary artery through carotid arterial access. The LM was engaged and coronary angiogram performed. Angiography was used to confirm flow to both the LAD and LCx. The IO or AAV particles were then slowly infused via a syringe directly attached to the end the diagnostic catheter over a 60–90 s period, and the catheter was subsequently flushed with 5 ml of saline to wash any excess IO or AAV remaining in the catheter.

### Delivery Technique: Balloon Occlusion Intracoronary Infusion (BO)

In both the IO and AAV study arms, four swine underwent delivery with the BO approach (Figure 2C, Supplementary Video 2). BO delivery was performed with standard coronary angioplasty techniques. After engaging the LM with a JR 4 coronary guide catheter (Cordis, Santa Clara, CA), a 0.014" 300 cm pre-curved guidewire was advanced into the LAD. A 2.50–2.75 mm × 12 mm balloon (Emerge OTW PTCA Dilation Catheter, Boston Scientific, Marlborough, MA, USA) with a distal infusion port was used to target the infusion to the distal LAD territory. To facilitate infusion through the coronary angioplasty catheter, an “Over-the-Wire” type balloon had to be used. The balloon was advanced over the guidewire into the mid-LAD, distal to the first large diagonal branch. As the guidewire was rapidly withdrawn by an assistant, the primary operator stabilized the balloon and quickly aspirated and flushed the lumen with 2–3 ml of heparinized saline to prevent clotting. The balloon was then inflated to nominal pressure and an angiogram was rapidly obtained to confirm occlusion of the vessel (Figure 2C). A syringe with 5 ml therapeutic was attached and slowly injected over a 60–90 s period. The catheter was then flushed with 3 ml of normal saline to deliver any remaining IO and the balloon was deflated. Full reperfusion was confirmed with an angiogram.

### Delivery Technique: Sidewall Intracoronary Infusion (SW)

Two swine had infusion via the SW technique in the IO study arm, but this technique was not utilized in the AAV arm (Figure 2D). The SW technique is very similar to the BO technique, using cannulation of the left main coronary artery to pass an 0.014" guidewire into the LAD. However, rather than an angioplasty balloon, a perforated sidewall balloon (ClearWay Rx, Atrium Medical, Somnotec, Ubi Techpark, Signapore) was passed over the wire. Five milliliter of therapeutic were delivered through an insufflation device at low pressures (2 atm) for 60–90 s. The catheter was then flushed with 3 ml of normal saline to deliver any remaining IO.

### IO Analysis: *Ex-vivo* Magnetic Resonance Imaging

All hearts were harvested via sternotomy within 15 min of IO delivery under deep anesthesia, after systemic administration of a high potassium chloride (KCl) solution to induce cardiac asystole. CMR imaging was performed immediately following harvest with fresh, unpreserved hearts. The harvested hearts were suspended in a container and the ventricles were filled with an MRI-inert fluid (Fomblin, Solvay Solexus). This allows the heart to maintain a diastolic shape with no signal from inside the ventricular chambers. The container was positioned in the center of a 64-channel head and neck coil and imaged using a 3T whole-body MRI scanner (Siemens Vida, Siemens Healthineers, Erlangen Germany). A whole-heart 3D inversion recovery (IR) sequence was obtained using the following parameters: TE/TR/flip angle: 2.03 ms/4.38 ms/15°, voxel size: 0.5 × 0.5 × 1.0 mm, and inversion time: 300 ms (set to suppress signal from remote myocardium). Scan time for each acquisition was 10–12 min.

The 3D whole-heart (IR) sequence was used to determine the presence and location of iron deposits within the myocardium. After reconstructing the 3D heart volume in a short-axis orientation, the left ventricle (LV) was divided into anterior, septal, lateral, and inferior regions. The anterior and inferior RV insertion sites were used to define the intraventricular septum. The posterior segment was defined as encompassing the inferior wall adjacent to the posterior papillary muscle, while the anterior wall was defined from the anterior insertion of the RV wall to the anterolateral papillary muscle. The lateral wall was defined as the section between the anterior and posterior papillary muscles. Within each section, iron accumulation (identified as signal voids within the myocardium due to iron accumulation in tissue) were manually planimetered. The volume of iron accumulation was calculated as a percent of overall LV volume and as percent of volume within each specific LV region.

### AAV Transduction Analysis: Cell Culture and Recombinant Virus Production

AAV9 production followed the same standardized approach for all experiments. Suspension-adapted HEK293 cells were cultured in F17 Expression Medium (Thermo Fisher A1383501) supplemented with 0.1% Pluronic F-68 (Millipore Sigma K4894), 4mM GlutaMAX (Thermo Fisher 35050061) and 1% penicillin-streptomycin (P-S) (Thermo Fisher 15070063) in a ReadyToProcess Wave 25 Rocker bioreactor (Cytiva 28988000).

Recombinant AAV vectors were produced using a triple plasmid transfection method consisting of an AAV *rep-cap* plasmid (AAV9 capsid variant), an adenoviral helper plasmid (pXX680), and a transgene packaging cassette, flanked by AAV2 inverted terminal repeat (ITR) sequences. Media supernatant was harvested 5–6 days post-transfection and subjected to tangential flow filtration (Repligen S02-E100-10-N) followed by PEG precipitation. Viral vectors were purified via iodixanol density gradient ultracentrifugation, followed by Dulbecco's phosphate-buffered saline (dPBS) containing 0.001% Pluronic-F68 buffer exchange using Pierce Protein Concentrator 100 kDa molecular weight cut-off (MWCO) spin columns (Thermo Fisher 88533). Titers of purified virus preparations were determined by quantitative PCR with primers amplifying the AAV2 ITR regions (forward, 5′ AACATGCTACGCAGAGAGGGAGTGG-3′; reverse, 5′-CATGAGACAAGGAACCCCTAGTGATGGAG-3′) (IDT Technologies, Ames IA).

### AAV Transduction Analysis: Screening for Neutralizing Antibodies

Prior to vector delivery, all animals in the AAV study arm were screened for neutralizing antibodies using previously described methods (16). Specifically, 25 μL of porcine serum was mixed with an equal volume containing recombinant AAV9 (1,000–10,000 vg/cell) packaging ssCBA-Luc transgenes prediluted in DMEM + 5% FBS + penicillin-streptomycin. The mixture was added to tissue culture-treated, black, glass- bottom 96-well plates (Corning) and incubated at room temperature for 30 min. A total of 5 × 10^4^ HEK293 cells in 50 μL of medium was then added to each well, and the plates were incubated in 5% CO_2_ at 37°C for 48 h. Cells were subsequently lysed with 25 μL of 1× passive lysis buffer (Promega) for 30 min at room temperature. Luciferase activity was measured on a Victor 3 multilabel plate reader (PerkinElmer) immediately after the addition of 25 μL of luciferin (Promega). All readouts were normalized to controls with no serum treatment. The ssCBA-Luc AAV was only used for the neutralizing antibody screen and not for *in vivo* delivery.

### AAV Transduction Analysis: Tissue Preservation and Processing

AAV vectors were delivered at doses of 4 × 10^13^ to 1 × 10^14^ of viral particles (vp) per animal. At 4–6 weeks post-AAV delivery, hearts were harvested immediately following cardiac arrest with systemic KCl administration and both coronary arteries were perfused with 500 mL of heparinized cold cardioplegic solution (Plegisol, Pfizer, New York, USA) using a 7-french catheter. Biopsies were systematically taken from 16 pre-specified locations (Supplementary Figure 1) from both ventricles and immediately snap-frozen in liquid nitrogen for subsequent viral DNA extraction.

### AAV Transduction Analysis: Viral Genome Quantification

Frozen biopsies were subjected to genomic DNA isolation using a Purelink Genomic DNA isolation kit. ITR primers were used to quantify viral genome copies via qPCR with 10 ng gDNA loaded per reaction. A standard curve was generated using viral vector plasmid with known concentration. Three separate replicates from each of the 16 biopsy locations were analyzed. Viral tissue transduction was then presented as average mass of viral DNA (vp/μg gDNA) grouping the biopsy locations into different regions of the heart including the LV lateral wall, LV anterior wall, LV apex, and RV.

### AAV Transduction Analysis: At Risk Region

Porcine models of myocardial infarction are commonly utilized to model acute injury as well as ischemic cardiomyopathy and study candidate therapeutics. The left anterior descending (LAD) coronary artery is the most commonly utilized target for such models, including parallel studies at our institution, due to fairly consistent perfusion territory and ease of exposure for surgical ligation or ease of access via coronary angioplasty techniques (17). With a mid-LAD occlusion technique, areas of ischemia include the distal interventricular septum, apical LV and, notably in porcine models, also includes the apical RV (18). Coronary angiography on pigs commonly demonstrates one or more large branches to the RV from the distal LAD (Supplementary Figure 2). As such, for comparisons of delivery techniques, the “At Risk Region” was defined as the distal septum and apical LV/RV, to parallel expected ischemic regions in a mid-LAD infarct model. Mean AAV transduction efficiency in the “At Risk Region” was compared to that from the “Non-Risk Region,” including the mid-anterior and lateral LV and RV free wall (Supplementary Figure 2).

### Statistical Analysis

For descriptive analyses, continuous variables were presented as mean ± standard deviation or median [interquartile range] depending upon the normality of distribution. Comparisons among the various delivery techniques were assessed with a global analysis of variance followed by pairwise Student's *t*-tests with Tukey adjustment for multiple comparisons. All statistics were performed using R Statistical Software (R Foundation for Statistical Computing, Vienna, Austria) and Prism (GraphPad Software, San Diego, California, USA).

## Results

### IO Retention Volume and Location

**Figure 3:**
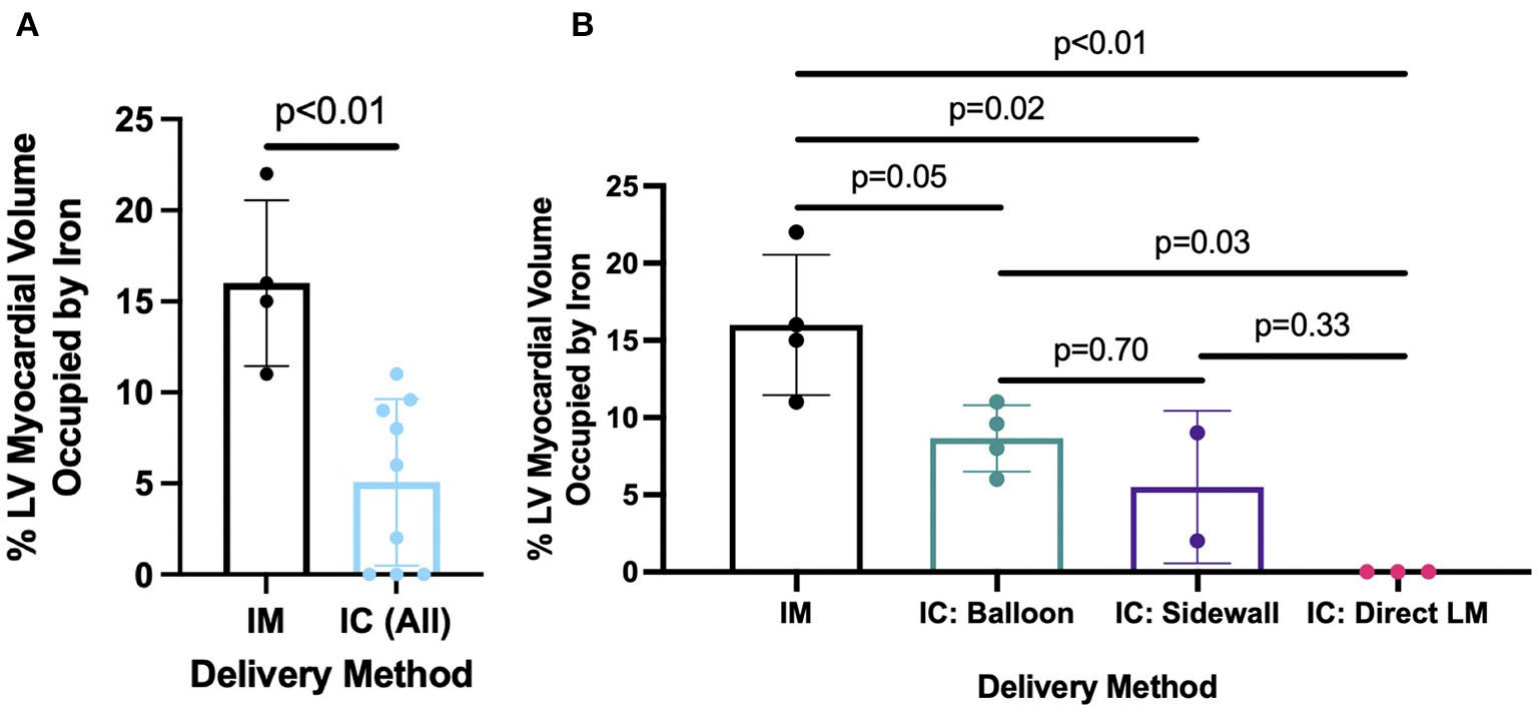
Percent of left ventricle occupied by iron oxide comparing **(A)** IM vs. combined IC delivery method and **(B)** IM vs. individual IC methods, including BO, SW, and LM.

**Figure 4:**
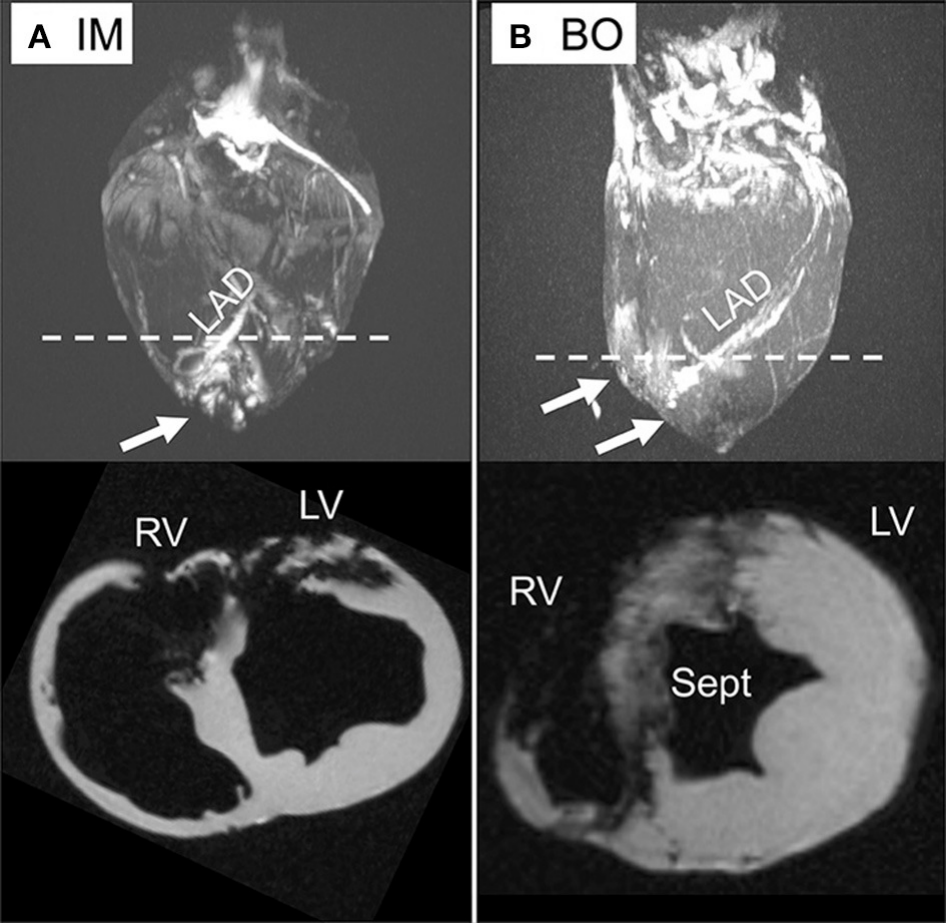
*Ex-vivo* CMR with 3D reconstruction and short axis views. **(A)** Direct intra-myocardial (IM) injection. **(B)** Intra-coronary balloon occlusion-distal infusion (BO) technique. Iron deposition is visible by bright regions in the 3D images (upper) and dark regions (signal void) in the short axis views (lower). While the IM technique has dense IO deposition in the apical left ventricle (LV) and anterior wall, the BO approach yields consistent delivery throughout the LV, septum (Sept), and right ventricle (RV), shown by arrows. The dotted line demonstrates the plane on 3D reconstruction corresponding to axial sections below.

**Figure 5:**
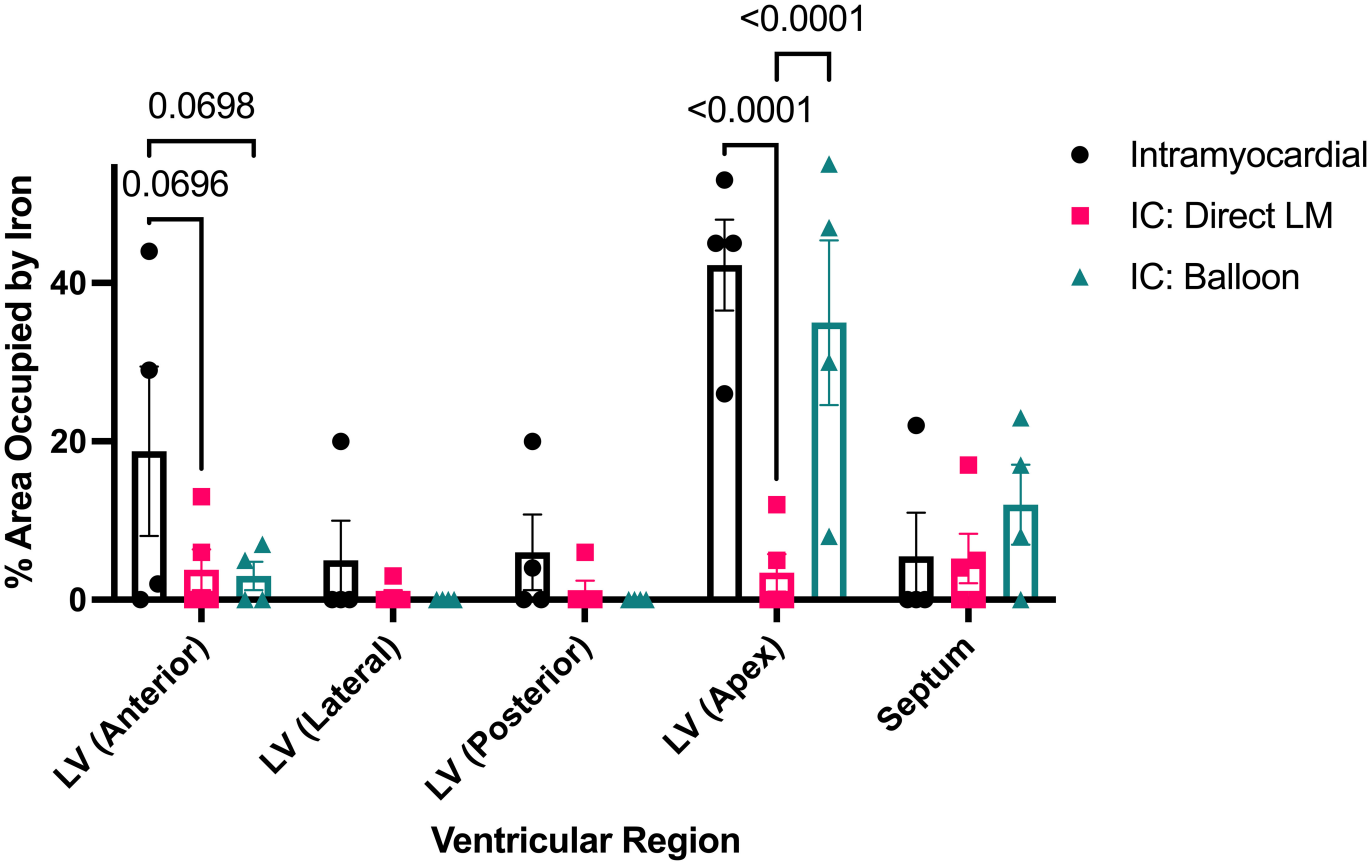
Spatial distribution of iron oxide shown by ventricular region and delivery method.

Delivery of 5 ml of IO nanoparticles was performed in 13 swine with the IM (*n* = 4), BO (*n* = 4), SW (*n* = 2), and direct LM (*n* = 3) techniques. On 3D CMR analysis, overall mean percent left ventricular volume of IO was 8.4 ± 6.8% with the various delivery approaches. The IM approach yielded significantly higher retention of IO compared to the combined IC approaches (16.0 ± 4.6 vs. 5.1 ± 4.6%, *p* = 0.008) (Figure 3). Within the IC approaches, BO had the highest retention of IO (8.7 ± 2.2%) as compared to SW (5.5 ± 4.9%) and LM (0%) (Figure 3). Assessing the territory of the IO retention qualitatively (Figure 4), IM delivery was limited to the anterior wall and apex, the regions directly accessible via this transepicardial approach, with minimal IO identified in the septum. By contrast, BO yielded consistent uptake throughout the distal LAD territory, including the septum, apical LV and, unique to porcine hearts, the apical RV (Figure 4, Supplementary Video 3). Quantitative assessment of IO retention by LV region (Figure 5) confirmed that both the IM and BO approaches yielded high uptake in the apex. IM generated higher uptake in the anterior wall than BO and BO yielded higher uptake in the septum than IM, although these differences were not statistically significant.

### Myocardial Injury

Based on pre- and post-operative troponin I levels in the IO substudy animals, IM delivery resulted in significantly greater myocardial injury with median increase in troponin I of 12,200 ng/L [IQR 6,240–15,600] compared to 158 ng/L [IQR 0–201] with IC delivery (*p* = 0.03) (Supplementary Figure 3). The two delivery methods with greatest concern for myocardial injury were the IM approach due to potential mechanical damage from needle penetration into the heart muscle (19) and the BO approach due to temporary (90 s) LAD occlusion, potentially resulting in ischemic damage. The LM approach did not involve the use of needle or transient ischemia and was not assessed. Directly comparing the IM and BO methods, the degree of myocardial injury was greater with IM compared to BO [increase in troponin I 153 [IQR 23–374]], although this difference did not reach statistical significance (*p* = 0.11).

### Therapeutic Model: Long-Term Viral Tissue Transduction

**Figure 6:**
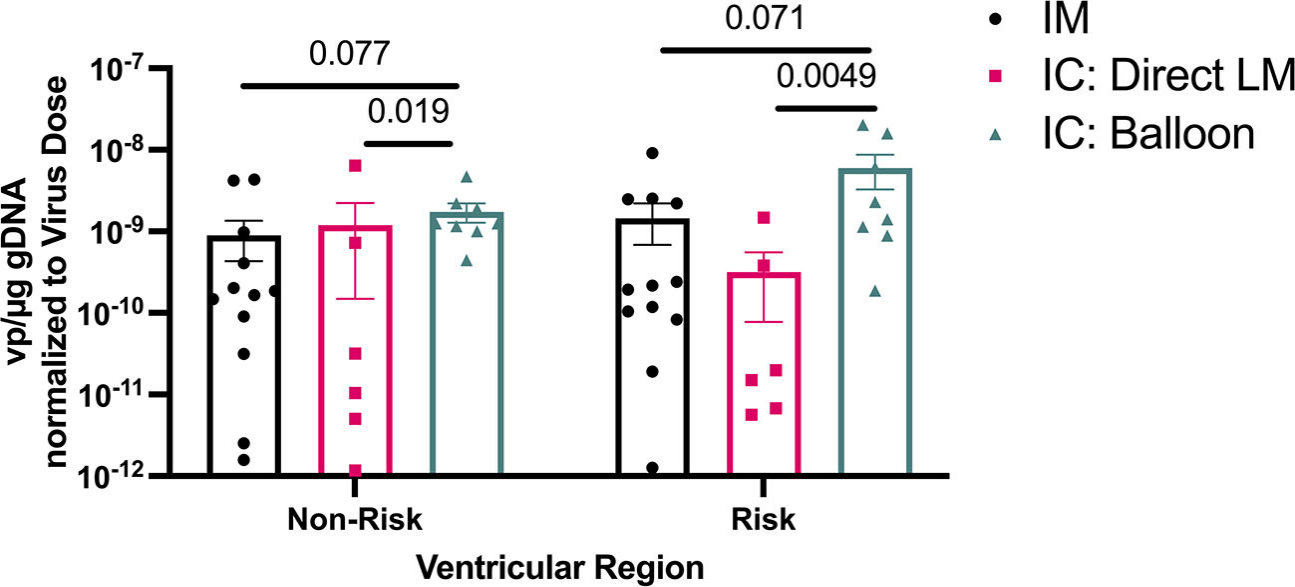
Viral DNA amounts in ventricular regions at risk and non-risk in an LAD infarct model using different AAV delivery methods. Note use of logarithmic axis.

In parallel experiments, an adeno-associated virus (AAV) vector was delivered via IM (*n* = 6), BO (*n* = 4), and LM (*n* = 3) techniques in a survival model. The BO approach achieved the highest tissue transduction of virus in the targeted territories, particularly the LV apex (BO = 5.3 × 10^−9^, IM = 1.8 × 10^−9^, LM = 5.0 × 10^−10^ viral copies per μg genomic DNA), although these differences did not reach statistical significance. All three techniques resulted in relatively lower transduction in non-targeted regions, such as the lateral LV free wall supplied by the left circumflex artery (BO = 1.0 × 10^−10^, IM = 9.2 × 10^−10^, LM = 1.9 × 10^−10^). Dividing the heart into “At Risk” and “Non-Risk” territories, the BO approach yielded the highest uptake in the “At Risk” region (*p* = 0.005 BO vs. LM, *p* = 0.071 BO vs. IM, Figure 6), although location of IM injection in LV was selected based on the expected downstream territory of the LAD.

## Discussion

The optimal delivery routes for cardiac cell and gene therapies, such as AAV-based gene therapies, remain unclear. We conducted a two-phased study in a porcine model to investigate the acute retention of IO nanoparticles, comparable in size to AAV (25 nm), using CMR and long-term vector expression following AAV injection/infusion using qPCR. We showed that direct IM injection resulted in the highest volume retention of IO nanoparticles in the heart, while targeted BO approach was the most effective among the catheter-based approaches. Compared to IM injection, the less invasive catheter-based IC delivery resulted in improved viral uptake throughout the vascular territory of interest (i.e., potential “At Risk” region of an infarct) and significantly less myocardial injury measured by post-operative troponin I levels. Regarding the general agreement between acute IO retention and long-term AAV expression, initial distribution of delivered therapeutics throughout the heart is expected to be an important determinant of long-term tissue targeting and therapeutic outcomes.

A number of variables must be considered when assessing the benefits and limitations of studied cardiac delivery techniques. For example, IM delivery suffers from non-uniform distribution of therapeutics near the injection sites, as shown by White et al. who demonstrated small heterogeneous pockets of AAV expression at myocardial depth corresponding to needle length (20). IC delivery, on the other hand, is limited by the ability of therapeutics to penetrate endothelial barrier of coronary vessels during short circulation time through the heart. Increased dwell time has been proposed as a first step to address this barrier (21), which was achieved in the current study with the balloon occlusion technique. The delivery may be also improved by co-infusion of nitroglycerin or histamine to increase vascular permeability (22) or simultaneous occlusion of venous outflow from the vascular bed of interest (6).

The differential regenerative potential of various regions of myocardium post-infarct highlights the importance of targeted therapeutic delivery. Prior studies have identified the infarct border zone (BZ), the perfused but hypocontractile region bordering the infarct, as the transcriptomically distinct region in zebrafish, mice, pigs, and humans (23–25). This region also shows the highest cardiomyocyte proliferative response to injury (26–28). Therefore, optimizing delivery to and viral transduction within the infarct BZ may be of high therapeutic significance for post-MI disease. As demonstrated in this study, while IM delivery yielded highest volume retention in the heart, this volume was concentrated in areas most visible and accessible from the epicardial surface. The BZ, however, represents a three-dimensional volume surrounding the infarct with spatial distribution that is highly dependent upon anatomy of coronary arteries and collateral vessels. As such, targeting the entire BZ with IM delivery is likely unfeasible, as was evident in this study from the absence of IO particles in the septum, a myocardial region highly affected by LAD infarct. In contrast, IC techniques allow distributed delivery throughout the entire affected region, with BO delivery allowing focused targeting to the exact coronary arterial bed affected by an infarct (i.e., the interventricular septum, anterior LV wall, apex, and RV in swine), not achieved by a less efficient LM delivery.

A thoracotomy approach to deliver therapeutics via IM injection (29, 30) (i.e., right thoracotomy in current porcine model and left anterior thoracotomy in humans) carries associated risks and morbidity and could benefit from less invasive procedures involving smaller incisions with less rib spreading, the primary mediator of post-thoracotomy pain (31–33). Moreover, in patients with adhesions from prior cardiac surgery (e.g., coronary artery bypass grafting), certain regions of the heart may be completely inaccessible, which would preclude significant manipulation required to expose the areas of interest and perform IM injection. The potential impact of myocardial damage from direct needle infiltration during IM injection must also be considered. In the current study, the IM approach was associated with significantly higher post-operative troponin I levels, despite the use of the smallest feasible needle size (25-gauge) (19). While Posvic et al. found no significant relationship between troponin I elevation and long-term major adverse cardiovascular events (34), there have been multiple reports of fatal tachyarrhythmias immediately after direct IM injection of stem cells in humans (35, 36). The exact underlying mechanisms are unknown, but needle injury along with resulting myocardial edema may contributed to the observed electrical instability. Thus, the risk of ventricular arrhythmias is another important area of consideration when deciding on the optimal method to deliver therapeutics to the heart.

This study has multiple limitations inherent to the study design. First, while animals were of similar weights and ages, the physical heart size may be variable, which could affect the measurements of percent of LV volume occupied on CMR. Second, as the iron oxide studies were acute, long-term implications of direct needle injury during IM injection could not be assessed. Third, since the AAV experiments involved delivery of multiple target genes, beyond the scope of this study, the analysis of data presented was limited to the efficacy of viral transduction in different heart regions, rather than expression of delivered transgene. Finally, while IO and AAV are nearly identical in size (25 nm), uptake of these molecules differ in the AAV is receptor dependent. Thus, mechanisms beyond just delivery technique may play a role in uptake through the endothelium into the interstitial space.

In summary, with ongoing evolution of transcatheter approaches for coronary arterial and valvular diseases, it is essential to establish techniques that can deliver therapeutics without (re)entering the chest and targeted to a specific region of interest. In this study, despite lower retention than IM injection, less invasive IC techniques, particularly paired with proximal balloon occlusion to limit competitive flow, resulted in efficient uptake of both AAV and similarly sized nanoparticles. BO also allowed for delivery to the entire vascular bed of interest and regions of the heart not easily reached via epicardial IM injection. Further investigations in large animal models to understand the implications of each technique for AAV-based therapeutic outcomes are warranted.

## Data Availability Statement

The raw data supporting the conclusions of this article will be made available by the authors, without undue reservation.

## Ethics Statement

The animal study was reviewed and approved by Duke University Institutional Animal Care and Use Committee (IACUC).

## Author Contributions

AV, SD, and AW: study design, study execution, data analysis, and manuscript drafting and revision. DW: study design, study execution, and manuscript drafting and revision. YC and DB: study design, study execution, and manuscript revision. RY, MB, GD, AA, and KP: study design and manuscript revision. NB: study design, study execution, data analysis, and manuscript revision. All authors contributed to the article and approved the submitted version.

## Funding

This work was supported by Translating Duke Health Initiative from Duke University and National Institutes of Health grants U01HL134764 to NB and 5T32HL069749 to AV.

## Conflict of Interest

The authors declare that the research was conducted in the absence of any commercial or financial relationships that could be construed as a potential conflict of interest.

## Publisher's Note

All claims expressed in this article are solely those of the authors and do not necessarily represent those of their affiliated organizations, or those of the publisher, the editors and the reviewers. Any product that may be evaluated in this article, or claim that may be made by its manufacturer, is not guaranteed or endorsed by the publisher.

## Supplementary Material

Supplementary Video 1 Cineangiographic images of left main (LM) coronary angiogram demonstrating territory perfused with direct LM infusion techniques and large arterial branch from the distal LAD to the apical RV, an anatomic finding commonly observed in pigs but not in humans.

Supplementary Video 2 Cineangiographic images of balloon occlusion (BO) of the mid left anterior descending (LAD) coronary artery. After confirming complete occlusion of the vessel on angiogram, the wire is quickly removed to facilitate infusion of therapeutic. Occlusion time is 60-90 seconds.

Supplementary Video 3 3D reconstruction of cardiac MRI after delivery of iron oxide via BO technique. There is significant signal void (bright in this sequence) throughout the apical RV, LV and septum.

Supplementary Figure 1 Pre-specified biopsy locations in the ventricular myocardium for AAV experiments with colors indicating groupings based on region.

Supplementary Figure 2 “At Risk Region” and pig coronary anatomy for a mid-LAD infarct model. **(A,B)** Representative gross specimen **(A)** and cardiac MRI **(B)**, with the perfusion territory for the distal LAD highlighted in orange and occlusion point for a mid-LAD infarct model represented by the blue square. **(C)** Representative coronary angiography showing a large branch (arrow) from the distal LAD to the RV that is commonly observed in pigs. LM, left main coronary artery; LCx, left circumflex coronary artery; LAD, left anterior descending coronary artery; LV, left ventricle; RV, right ventricle.

Supplementary Figure 3 Change in troponin I level pre- vs. post-delivery of IO after IM vs. combined IC delivery methods.

## Abbreviations

BO: Balloon Occlusion-Distal Infusion Technique
BZ: Border Zone
IC: Intra-coronary
IM: Intra-myocardial
IO: Iron oxide
LM: Direct Left Main Infusion Technique
SW: Sidewall Catheter Infusion Technique.
