# Supplementary figures and images for: Targeted Delivery for Cardiac Regeneration: Comparison of Intra-coronary Infusion and Intra-myocardial Injection in Porcine Hearts
Source: Front Cardiovasc Med. 2022 Feb 10;9:833335. doi: 10.3389/fcvm.2022.833335 (PMC8866722; doi:10.3389/fcvm.2022.833335)

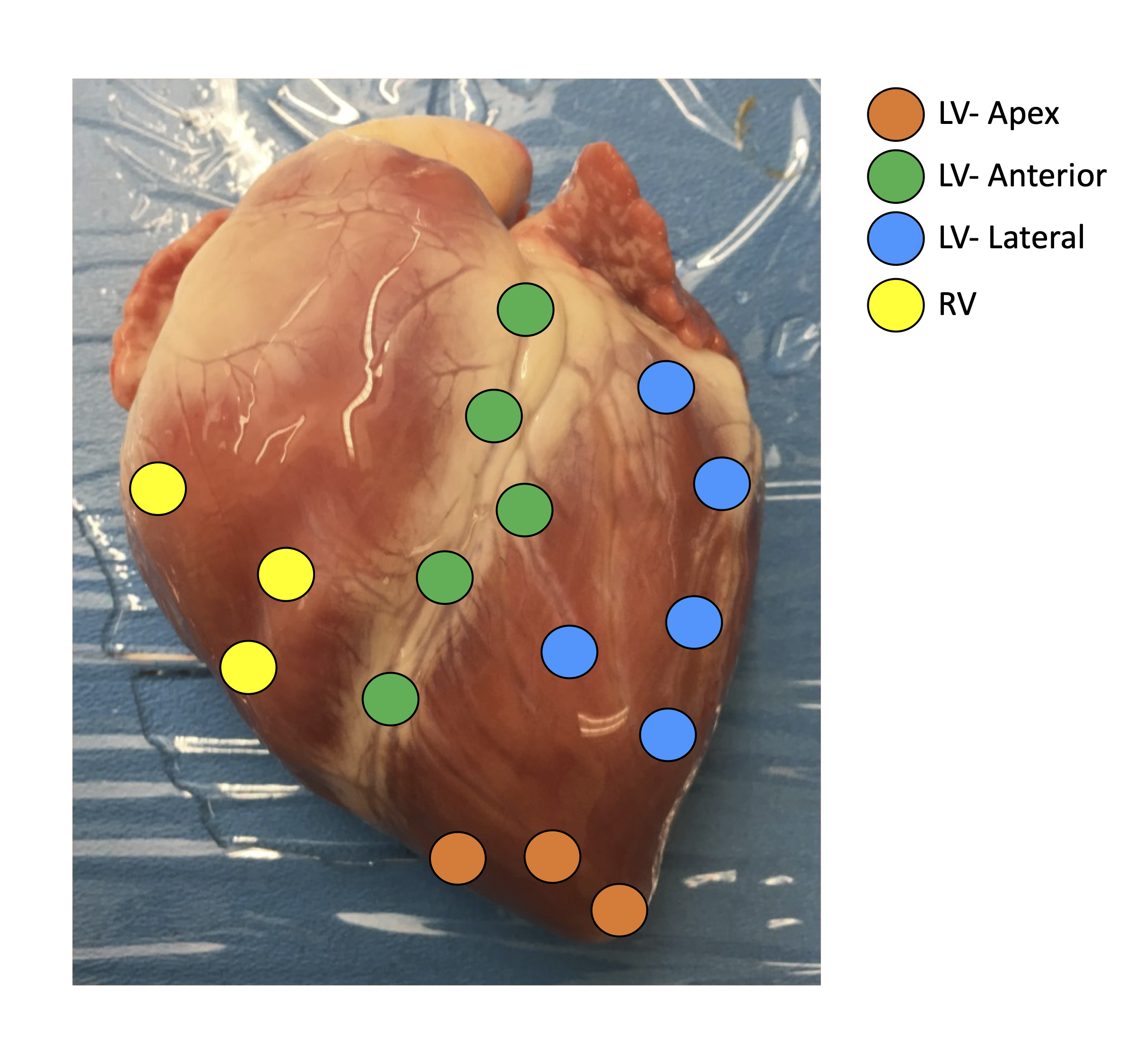

Supplement: Supplementary Figure 1 — Pre-specified biopsy locations in the ventricular myocardium for AAV experiments with colors indicating groupings based on region. [file Data_Sheet_1.ZIP › Vekstein Rev Supp Figures/Supplemental Figure 1.tiff]

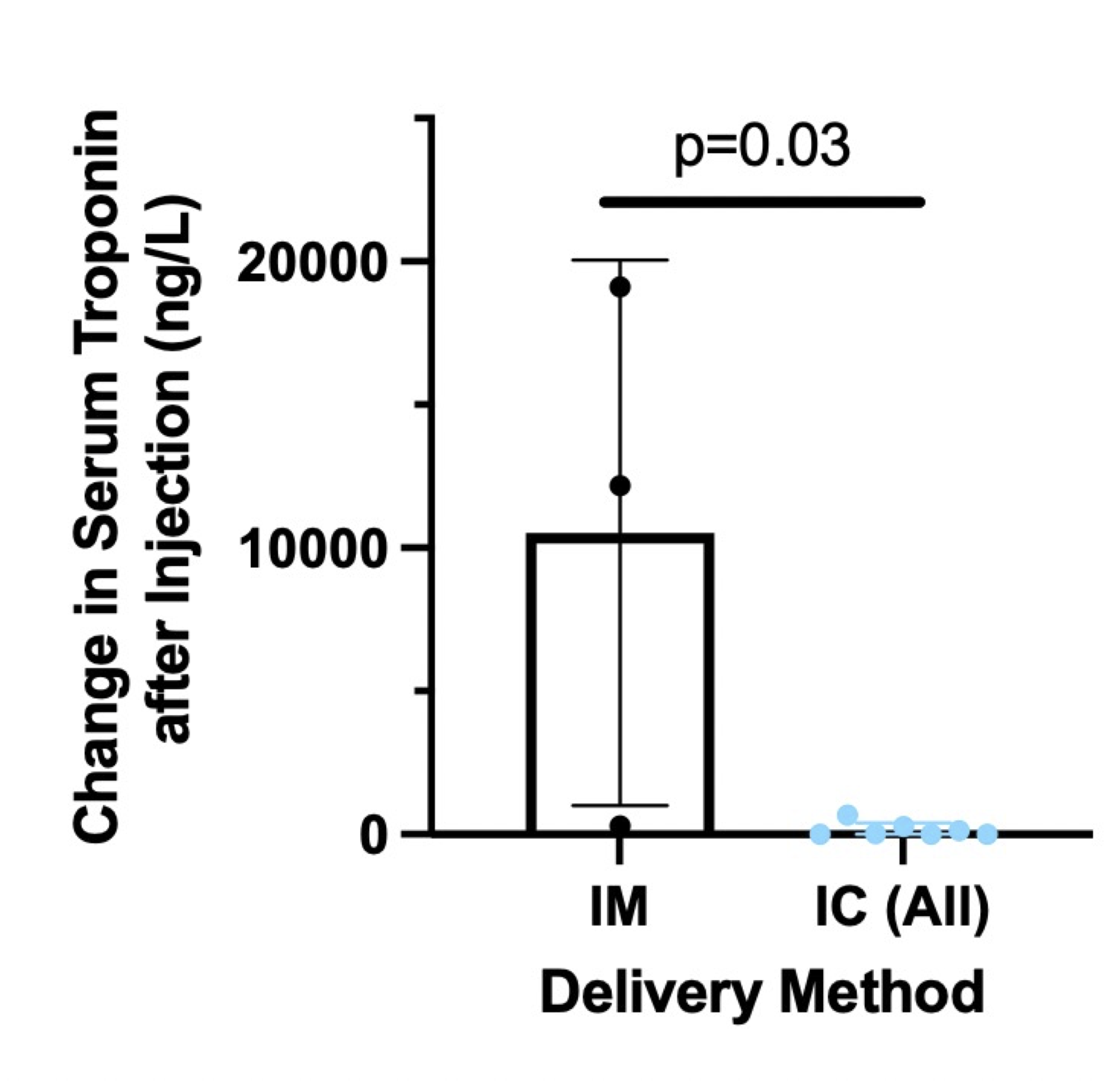

Supplement: Supplementary Figure 1 — Pre-specified biopsy locations in the ventricular myocardium for AAV experiments with colors indicating groupings based on region. [file Data_Sheet_1.ZIP › Vekstein Rev Supp Figures/Supplemental Figure 3.tiff]

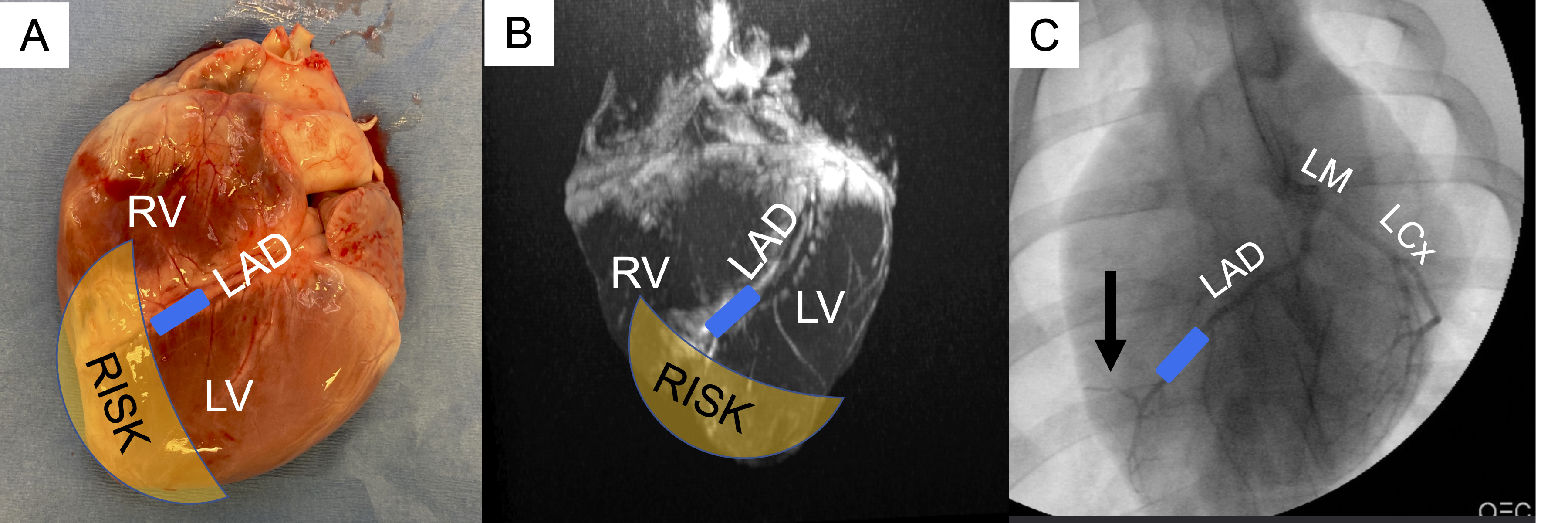

Supplement: Supplementary Figure 1 — Pre-specified biopsy locations in the ventricular myocardium for AAV experiments with colors indicating groupings based on region. [file Data_Sheet_1.ZIP › Vekstein Rev Supp Figures/Supplemental Figure 2.tiff]
